# Etiologic Diagnosis of Lower Respiratory Tract Bacterial Infections Using Sputum Samples and Quantitative Loop-Mediated Isothermal Amplification

**DOI:** 10.1371/journal.pone.0038743

**Published:** 2012-06-14

**Authors:** Yu Kang, Rui Deng, Can Wang, Tao Deng, Peichao Peng, Xiaoxing Cheng, Guoqing Wang, Minping Qian, Huafang Gao, Bei Han, Yusheng Chen, Yinghui Hu, Rong Geng, Chengping Hu, Wei Zhang, Jingping Yang, Huanying Wan, Qin Yu, Liping Wei, Jiashu Li, Guizhen Tian, Qiuyue Wang, Ke Hu, Siqin Wang, Ruiqin Wang, Juan Du, Bei He, Jianjun Ma, Xiaoning Zhong, Lan Mu, Shaoxi Cai, Xiangdong Zhu, Wanli Xing, Jun Yu, Minghua Deng, Zhancheng Gao

**Affiliations:** 1 Department of Respiratory and Critical Care Medicine, Peking University People’s Hospital, Beijing, People’s Republic of China; 2 Key Laboratory of Genome Sciences and Information, Beijing Institute of Genomics, Chinese Academy of Science, Beijing, People’s Republic of China; 3 Department of Biomedical Engineering, School of Medicine, Tsinghua University, Beijing, People’s Republic of China; 4 School of Mathematical Sciences and Center for Theoretical Biology, Peking University, Beijing, People’s Republic of China; 5 Department of Assay Development, Capitalbio Corporation, Beijing, People’s Republic of China; 6 Department of Respiratory Medicine, Fujian Provincial Hospital, Fuzhou, People’s Republic of China; 7 Department of Respiratory Medicine, Beijing Children’s Hospital Affiliated to Capital Medical University, Beijing, People’s Republic of China; 8 Department of Emergency Medicine, Beijing Children’s Hospital affiliated to Capital Medical University, Beijing, People’s Republic of China; 9 Department of Respiratory Medicine, Xiangya Hospital of Central South University, Changsha, People’s Republic of China; 10 Department of Respiratory Medicine, The First Affiliated Hospital of Nanchang University, Nanchang, People’s Republic of China; 11 Department of Respiratory and Critical Care Medicine, The Third Affiliated Hospital of the Inner Mongolia Medical College, Baotou, People’s Republic of China; 12 Department of Respiratory Medicine, Ruijin Hospital Affiliated to Shanghai Jiaotong University, Shanghai, People’s Republic of China; 13 Department of Respiratory Medicine, The First Hospital of Lan Zhou University, Lanzhou, People’s Republic of China; 14 Department of Respiratory Medicine, The Third Affiliated Hospital of Guangzhou Medical College, Guangzhou, People’s Republic of China; 15 Department of Respiratory Medicine, Lianyungang First People’s Hospital affiliated to Xuzhou Medical College, Lianyungang, People’s Republic of China; 16 Department of Respiratory Medicine, NO.263 Clinical Section of the Military General Hospital of Beijing, Beijing, People’s Republic of China; 17 Institute of Respiratory Disease, The First Affiliated Hospital of China Medical University, Shenyang, People’s Republic of China; 18 Department of Respiratory Medicine, Renmin Hospital of Wuhan University, Wuhan, People’s Republic of China; 19 Department of Respiratory Medicine, Henan Province People’ Hospital, Zhengzhou, People’s Republic of China; 20 Department of Respiratory Medicine, The First Affiliated Hospital of Tsinghua University, Beijing, People’s Republic of China; 21 Department of Respiratory Medicine, The Affiliated Hospital of GuiYang Medical College, Guiyang, People’s Republic of China; 22 Department of Respiratory Medicine, Peking University Third Hospital, Beijing, People’s Republic of China; 23 Department of Respiratory Medicine, Lanzhou Pulmonary Hospital, Lanzhou, People’s Republic of China; 24 Department of Respiratory Medicine, The First Affiliated Hospital of Guangxi Medical University, Nanning, People’s Republic of China; 25 Department of Respiratory Medicine, Peking University Space Central Hospital, Beijing, People’s Republic of China; 26 Department of Respiratory Medicine, Nanfang Hospital Affiliated to Southern Medical University, Guangzhou, People’s Republic of China; 27 Section of Pulmonary and Critical Care Medicine (MC 6076), Department of Medicine, University of Chicago, Chicago, Illinois, United States of America; University of Calgary & ProvLab Alberta, Canada

## Abstract

Etiologic diagnoses of lower respiratory tract infections (LRTI) have been relying primarily on bacterial cultures that often fail to return useful results in time. Although DNA-based assays are more sensitive than bacterial cultures in detecting pathogens, the molecular results are often inconsistent and challenged by doubts on false positives, such as those due to system- and environment-derived contaminations. Here we report a nationwide cohort study on 2986 suspected LRTI patients across P. R. China. We compared the performance of a DNA-based assay qLAMP (quantitative Loop-mediated isothermal AMPlification) with that of standard bacterial cultures in detecting a panel of eight common respiratory bacterial pathogens from sputum samples. Our qLAMP assay detects the panel of pathogens in 1047(69.28%) patients from 1533 qualified patients at the end. We found that the bacterial titer quantified based on qLAMP is a predictor of probability that the bacterium in the sample can be detected in culture assay. The relatedness of the two assays fits a logistic regression curve. We used a piecewise linear function to define breakpoints where latent pathogen abruptly change its competitive relationship with others in the panel. These breakpoints, where pathogens start to propagate abnormally, are used as cutoffs to eliminate the influence of contaminations from normal flora. With help of the cutoffs derived from statistical analysis, we are able to identify causative pathogens in 750 (48.92%) patients from qualified patients. In conclusion, qLAMP is a reliable method in quantifying bacterial titer. Despite the fact that there are always latent bacteria contaminated in sputum samples, we can identify causative pathogens based on cutoffs derived from statistical analysis of competitive relationship.

**Trial Registration:**

ClinicalTrials.gov NCT00567827

## Introduction

Lower respiratory tract infections (LRTI) represent a major public health problem, accounting for 12% and 16% of inpatient hospitalization in urban and rural hospitals, respectively, in the People’s Republic of China. [1] Since traditional bacterial cultures for etiologic diagnoses of LRTI take at least 48 to72 hrs to complete and frequently produce false-negative results, the treatment of LRTI is often imprecise, and mistreatment contributes significantly to antibiotic misuse and overuse. [2], [3].

DNA- based methods (including indirectly for RNA) have been successfully applied in single or multiple pathogen detection, including those for TB, CMV, HIV, *Pneumocystis jirovecii*, etc. [4], [5] However, DNA-based methods for the identification of causative pathogens in sputa as routine diagnoses of LRTI have been hampered by inconsistent results with culture-based methods.[6]–[8] Discrepancies between the two methods have to be thoroughly explained before DNA-based methods can be routinely applied in LRTI diagnosis. Here, we report an application of a DNA-based assay– qLAMP (quantitative Loop-mediated isothermal AMPlification) [9] –in the detection of a panel of eight common bacterial pathogens in sputa of LRTI patients and provide an evaluation on the reliability of qLAMP in a close comparison to culture-based assays.

## Methods

The protocol for this trial and supporting CONSORT checklist are available as supporting information; see Checklist S1 and Protocol S1.

### Patients

The suspected LRTI patients diagnosed by physicians in 21 tertiary hospitals in 14 provinces of the People’s Republic of China (Figure S1) were consecutively enrolled in this study. They were initially diagnosed as suspected cases for bacterial LRTI, having typical characteristics of pneumonia or bronchitis, which were firmly inferred from chest X-rays and one of the following criteria: (1) fever>38.5°C, (2) peripheral white blood cell count (WBC)>10.0×10^9^/L and neutrophil % >60%, (3) exacerbated dyspnea, and (4) exacerbated sputum production or purulence. Patients diagnosed with non-infectious diseases or infection by non-bacterial pathogens (including viruses, fungi, and *pneumocystis*) or TB were subsequently excluded from the cohort.

The study was approved by the Institutional Review Board of Peking University People’s Hospital and registered at www.clinicaltrial.gov (registration ID: NCT00567827). Written consent was obtained from all patients prior to recruitment. All participating researchers were trained thoroughly on patient enrollment, sample collection, and clinical information entry.

### Sample and Information Collection

**Figure 1:**
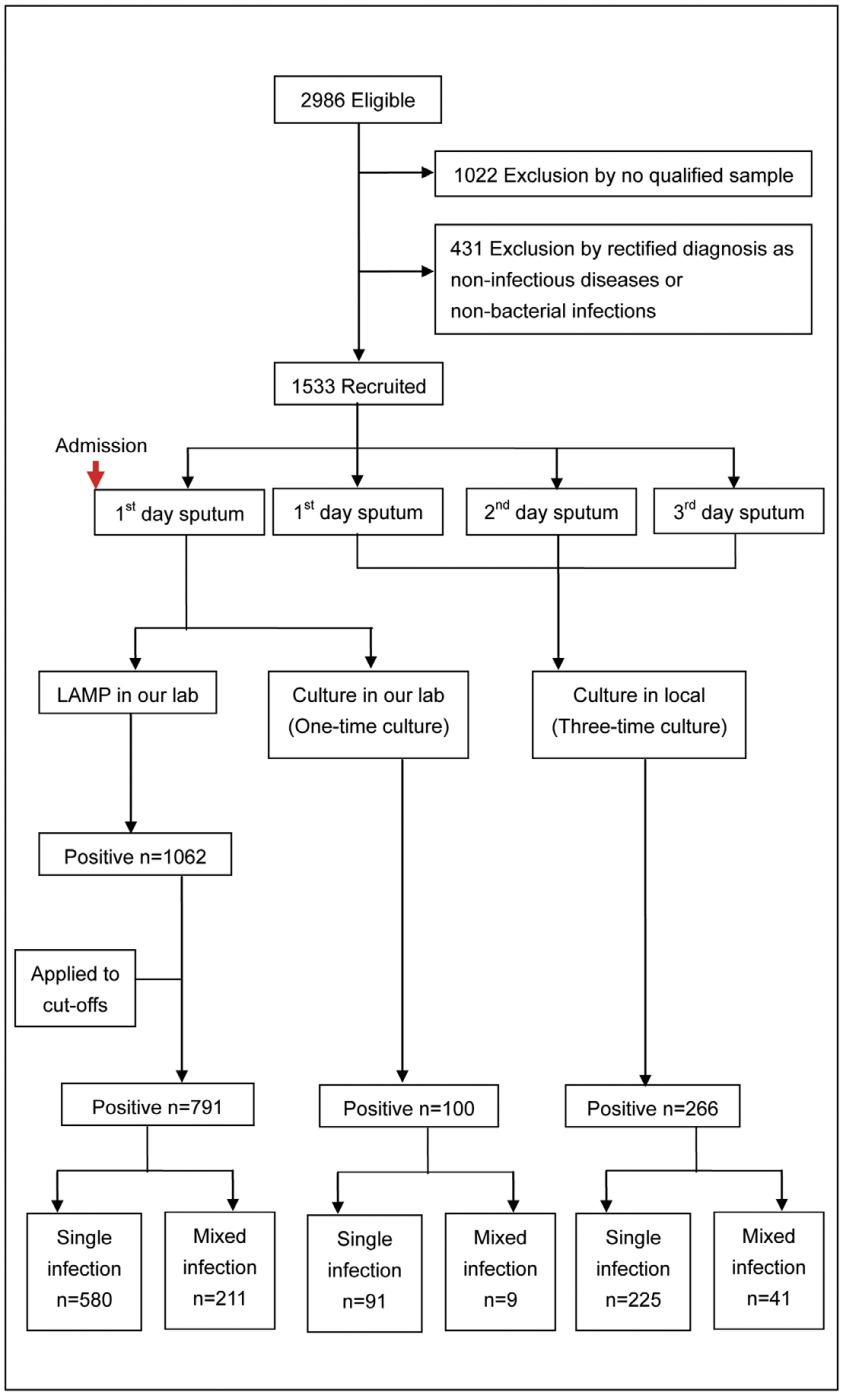
A flow chart of patient recruitment and sample processing. After admission, we collected 4 sputum samples from each patient. The same samples are used for the parallel qLAMP and culture tests. The results from qLAMP and one-time culture are from the same sample, and those of qLAMP and three-time culture are from the same patient, but not the same sample.

From each enrolled patient, we collected four spontaneous early-morning sputa: two samples on the 1st day and one each on the 2nd and 3rd days of post-admission. One 1^st^-day sputum sample was evaluated using light microscopy (WBC ≥25 and epithelia cells <10 per 100×field), and qualified sputa (50% broth glycerol added) were transported to the central laboratories at Peking University People’s Hospital on dry ice and used for routine culture and qLAMP assays. The other three collections were subjected to routine culturing and identification (VITEK2 system, bioMérieux Inc, France) at the local hospitals. In some cases involving toddlers or infants, sputa or BALF samples were obtained using fiberoptic bronchoscopy (performed according to clinical requirements) (See Figure 1). We also collected clinical data, including demographic features, diagnoses, results of culture assays, antibiotic treatments, treatment outcomes, and lengths of hospital stays, and entered the information into a customized database.

### Laboratory Methods

For each patient, we carried out a parallel study using both routine culture-based and qLAMP assays. Each sputum sample was liquefied in equal volume of 10% NaOH, and DNA was isolated by using the Universal Kit for Bacterial DNA Extraction (Capitalbio Corporation, P. R. China). We designed PCR primers for the eight bacterial pathogens based on genomic sequences retrieved from Genebank (Table S1), and they are: *Acinetobacter baumannii*, *Escherichia coli*, *Haemophilus influenzae*, *Klebsiella pneumoniae*, *Pseudomonas aeruginosa*, *Staphylococcus aureus*, *Stenotrophomonas maltophilia* and *Streptococcus pneumoniae*. Each primer set was designed based on a previously-described strategy (Table S2 and Figure S2). [10], [11] Sensitivity, specificity, and reproducibility of the primers were evaluated based on quantified DNA from 27 bacterial species (Table S3). qLAMP was performed based on a real-time fluorescence detection method. [12] The titer was quantified according to standard curves obtained from pre-quantified DNA templates (Methods S1) as copy number per milliliter sputum. Two experienced technicians who were not aware of the sample identities conducted the qLAMP tests and routine cultures independently in two separate laboratories (the central laboratories) at the Peking University People’s Hospital.

### Statistical Analysis

#### Evaluating the robustness of qLAMP assay

To evaluate the congruence of qLAMP and culture results, we constructed a contingency table and used Fisher’s exact and chi-square tests. For each pathogen, we estimated the probability of being positive in culture as a function of the titer quantified in qLAMP in terms of logistic regression (Methods S1).

#### Determining cutoffs for the pathogen panel

For each patient, species X is a “pathogen candidate" (PC) if it has the maximal titer within the panel, and then the *probability of being PC* (P-PC) represents the relative competitive potency of the species. Given the fact that there are errors in empirical quantification and possibilities of mixed infection, species with a titer near maximum in the panel is also regarded as PC if its difference to maximum is less than 0.5 in logarithmic scale. Since all bacterial species in the panel also dwell in airways of healthy persons or patients in a latent period, we are able to assume that they may experience three similar stages in becoming pathogenic: lag phase (being latent within the microflora), log phase (being competitive to other species when propagates abnormally), and dominant phase (being pathogenic when dominates in the microflora). By using piecewise linear regression in 1–3 fold zig-lines, we estimated P-PC or the probability of a bacterium to become a pathogen candidate as a function of the titer or bacterial load for each species. In the linear regression chart, the breakpoints between segments are what we call cutoffs that lie either between normal flora and potential pathogens–the lower cutoff, or between potential pathogens and definite pathogens–the upper cutoff. For species that did not converge well, we stratified all qualified patients based on their clinical records and medical knowledge.

## Results

### Data from Sputum Cultures

**Figure 2:**
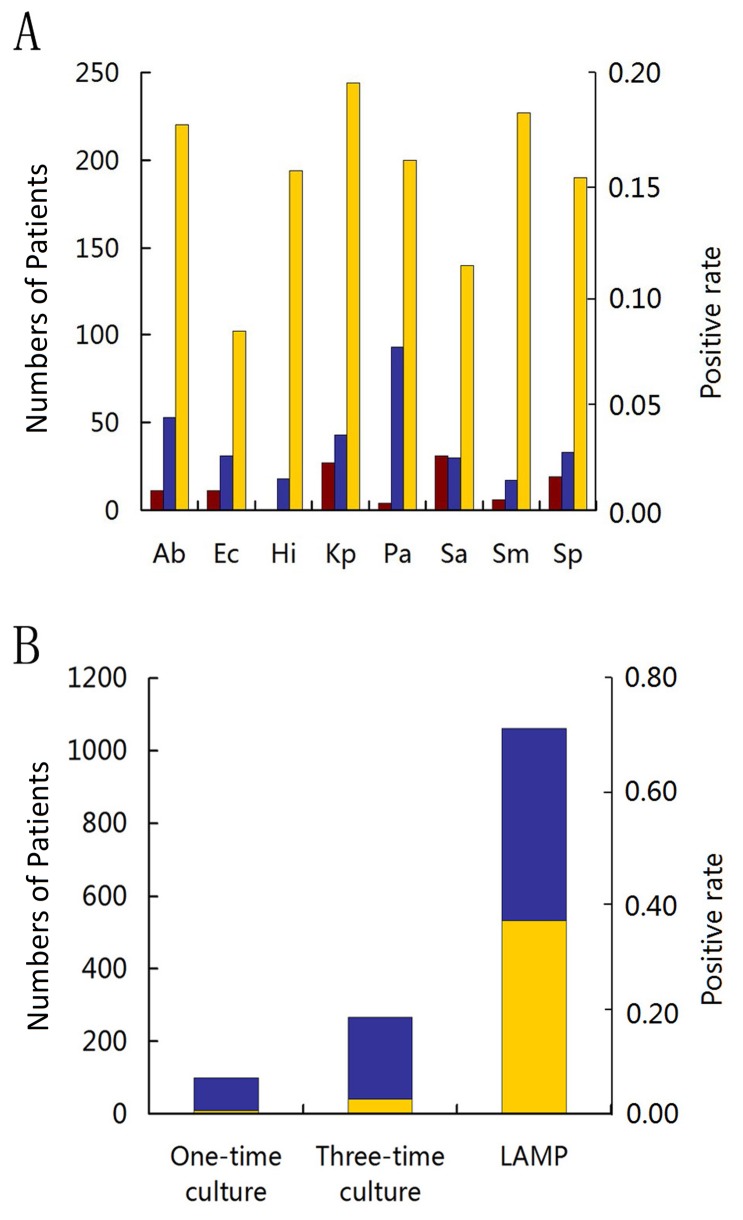
qLAMP and culture result from LRTI patients. (A) The positive rates (the right vertical axis) of one-time culture (brown bar), three-time culture (blue bar), and quantitative LAMP (yellow bar) for the eight species in the panel (from the left: Ab, *A. baumannii*; *Ec, E. coli;* Hi, *H. influenzae;* Kp, *K. pneumoniae;* Pa, *P. aeruginosa;* Sa, *S. aureus*; Sm, *S. maltophilia;* and Sp, *S. pneumoniae*) detected from the number of patients (the left vertical axis). (B) The number of patients (the left vertical axis) who were tested positive for at least one bacterium in one-time culture, three-time culture, and qLAMP. Each bar is the sum of patient with single (blue bar) and multiple (yellow bar) species detected.

We obtained raw data from 1533 inpatients out of 2986 eligible candidates qualified for the final analysis, based on samples collected from December 2007 to June 2009 (Figure 1). We summarized patient information including hospitals, demographic characteristics, and LRTI diagnoses in Tables S4 and S5. In culture assay, although we found 43 bacterial species and 17 fungi species in total, only the eight species in the pathogen panel are taken into account. For culture assays done in the central laboratories (one-time culture), we have 100 cases (6.52%) tested positive for at least one of the panel members, and for the culture done in local hospitals (three-time culture), we have 266 cases (17.35%) tested positive for at least one of the panel members in at least one of the three-time cultures (Figure 2). For each species, the average confirmation rates among all cultures (four times altogether: one done in the central laboratory and three done in local hospitals) range from 0.17 to 0.50 (Table 1). In other words, it is impossible to detect the same species of bacteria in a sputum sample in all repeated culture assays. The qLAMP and culture results from the central laboratories were never communicated to local hospitals, and the strategies of anti-infectious treatments are only based on the culture results obtained independently. No adverse effects are found either being caused by the protocols or as results of performing qLAMP and routine culture assays on patients during the study.

**Table 1:** Data Summary of qLAMP and culture assays.

	Ab		Ec		Hi		Kp		Pa		Sa		Sm		Sp	
qLAMP	+	−	+	−	+	−	+	−	+	−	+	−	+	−	+	−
	220	1313	102	1431	194	1339	244	1289	200	1333	140	1393	227	1306	190	1343
Total Culture	25	33	14	24	6	12	34	26	58	39	28	25	10	11	27	21
Three-time Culture	23	30	10	21	6	12	21	22	55	38	12	18	9	8	15	18
One-time Culture	6	5	7	4	0	0	22	5	3	1	24	7	3	3	16	3
ConfirmedCulture*	9	8	7	5	1	0	12	4	16	12	8	2	2	0	6	0
Confirmationrate**	0.36	0.24	0.5	0.208	0.17	0.00	0.35	0.154	0.27	0.307	0.285	0.08	0.2	0.00	0.222	0
Bacterial Mortality***	0.792		0.64		1.0		0.372		0.957		0.00		0.647		0.424	

Note: The Abbreviations are: Ab, *A. baumannii*; Ec, *E. coli*; Hi, *H. influenzae*; Kp, *K. pneumoniae*; Pa, *P. aeruginosa*; Sa, *S. aureus*; Sm, *S. maltophilia*; and Sp, *S. pneumonia*.

* indicates the number of patients whose positive culture was confirmed by one of the 4 culture-based tests.

** indicate confirmation rate of the positive cultures by one of the 4 culture-based tests.

*** indicate the bacterial mortality due to refrigeration, storage, and transportation.

### Data from qLAMP Assays

We optimized the primers to reach a sensitivity of 10^3^ copies/ml targeted DNA in sputa and high specificity that no detectable cross-reactions with control DNA from 26 other species of bacteria were found (Methods S1). For the clinical sputum data, we compared the qLAMP results to those of the cultures, and found that qLAMP assay is 2–11 times more sensitive when compared to the culture assays (three-time culture) (Figure 2A). In a total of 1062 patients (69.28% from all 1533 qualified patients), we detected at least one species positive in qLAMP; this result leads to sensitivities of 10.6 times when compared to those of the one-time culture and 4 times to those of the three-time culture (Figure 2B). Even though qLAMP is more sensitive than culture, we still have 0.6–2.8% of the positive cases suggested by the culture results but are unable to confirm them via qLAMP (Table 1).

### Relatedness between qLAMP and Culture Assays

**Figure 3:**
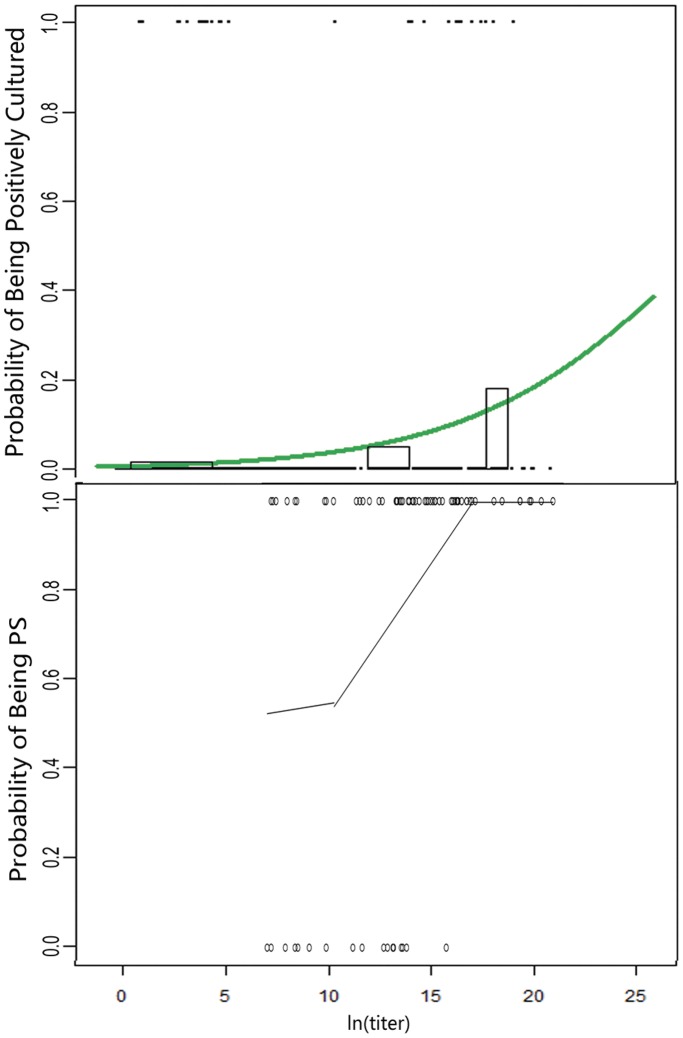
Examples of *S. pneumonia* showing the relationship between qLAMP and culture results (logistic regression) and cutoff determination based on competitive relationship (piece-wise linear regression). The horizontal axis displays the bacterial natural logarithmic titer in sputum sample. (A) Logistic regression curve (green line). Solid circles indicate patients; they are placed at the top of the chart when being test as positive and at the bottom of the chart when being tested as negative in the culture assays. The height and width of the bars display the frequency and the number of patients being tested positive in cultures, respectively. (B) Piecewise linear regression (black lines) of *S. pneumonia* in COPD patients. Open circles indicate patients; they are placed at the top of the chart when being PC (Pathogen Candidate) and at the bottom of the chart when NOT being PC.

We assess the relatedness between the two assays based on contingency table and logistical regression curve. For this part of the analysis, we only used the culture results from local hospitals, since there are variable bacterial mortalities (ranged 0–1 and averaged 0.604) detected in the central laboratories, largely due to refrigeration during the sample storage and transport periods. We made two observations. First, we noticed that the *p* values in contingency table, which evaluates independence between qLAMP and culture data, are extremely low overall. This suggests that the titers quantified via qLAMP are not stochastic and have a strong correlation to culture results (Table 2). Second, when we look at logistical regression for each bacterium, the probability of being positive in culture and the titer quantified via qLAMP fit logistical regression curves (Figure S3). As an example, we show a result of logistic regression for *S. pneumoniae* in Figure 3 A. The *p* values of logistic regression are all extremely low (Table 2) except for *H. influenzae* (*p* value 0.0115>0.01), mainly due to its fragility and low positive rate in culture. The strong correlations between the two methods based on the results of contingency table and logistic regression demonstrate the robustness of qLAMP assay.

**Table 2:** Relatedness between qLAMP and culture assays and cutoffs in different subgroups.

		Cutoff (copies/ml)		Contingency table	Logistic regression model	Positive rate under the titer of 10^3^copies/ml	
		Lower	Upper	*p* value of Independence between qLAMP and culture data	*p* value of ln(X)	Estimated	Actual
Ab	All	N	2.07×10^5^	1.92×10^−10^	1.08×10^−10^	0.0380	0.0229
Ec	All	N	6.67×10^4^	1.31×10^−14^	1.70×10^−8^	0.0282	0.0147
Hi	COPD	N	8.17×10^5^	7.90×10^−3^	1.15×10^−2^	0.0129	0.0090
	non-COPD	4.79×10^6^	N				
Kp	CAP	6.93×10^4^	N	1.34×10^−18^	3.30×10^−12^	0.0277	0.0171
	non-CAP	3.69×10^6^	N				
Pa	BX	N	3.33×10^6^	2.71×10^−45^	<2×10^−16^	0.0555	0.0285
	non-BX	1.55×10^5^	2.18×10^8^				
Sa	All	N	5.40×10^5^	2.62×10^−29^	1.84×10^−10^	0.0210	0.0129
Sm	Aged	N	1.47×10^5^	2.02×10^−5^	1.34×10^−4^	0.0113	0.0061
	non-Aged	1.13×10^6^	2.18×10^8^				
Sp	Children	N	1.19×10^4^ *	7.35×10^−21^	1.01×10^−9^	0.0219	0.0134
	COPD adults	2.15×10^4^	1.15×10^7^				
	non-COPD adults	1.62×10^5^	2.28×10^7^				

Note: We only used the data from the three-time culture to test the consistency between qLAMP and culture assays. N and BX stand for not found and bronchiectasis, respectively.

* In the piecewise linear regression of *S. pneumoniae* for child patients, all the detected titers were PCs (pathogen candidates), and no breakpoint was found. Therefore, the lowest titer detected in this subgroup is deemed as the upper cutoff.

Although the qLAMP method is more sensitive than the culture-based method, there are exceptional cases of positive results in culture that are not confirmed by qLAMP. However, these cases can also be explained by the relatedness between the two methods in term of logistical regression. When the titer or presence of the panel members is actually lower than the qLAMP detection limit of 10^3^ copies/ml, it is still possible that these exceptional positive cases in culture are contributed by several viable bacterial cells in samples that have a very low possibility or even can never be detected via qLAMP. When deducing the logistic regression curve to titer range below 10^3^ copies/ml, positive rate of culture can be estimated by calculation based on regression function, and our confidence is supported by the fact that the observed positive rate of culture assays is always lower than the estimated rate when titers are over-estimated as normal distribution within the range (Table 2).

### The Probability of being Pathogen Candidates (PC) and Deduced Cutoffs

When choosing piecewise linear regression to analyze our data, we found that for some species in the panel the curves converge when data of total patients are calculated, while for the others in the panel the curves only converge when patients are stratified in subgroups (cutoffs see Table 2, regression curves for each species see Figure S4). In the results from *H. influenzae*, *K. pneumoniae*, *P. aeruginosa*, *S. maltophilia*, and *S. pneumoniae*, we observed that covariates often play an important role, which can lead to different cutoffs in different subgroups, and that strategies of stratification based on medical knowledge improve convergence significantly. An example of *S. pneumoniae* in adult patients with COPD (chronic obstructive pulmonary disease) is shown in Figure 3B. The zig-like curve shows the probability of being PC (or competitive potency) for a bacterium that often experiences lag, log, and dominant phases during the process of becoming pathogenic status.

In a list of cutoffs derived from piecewise linear regression, there is a decrease in cutoff values for pathogens defined in subgroups of susceptible patients and such a decrease means that bacteria gain competitive advantages more easily in these patients (Table S6). For example, the cutoffs for *P. aeruginosa* are lower in the bronchiectasis patients than in other patients. Similarly, the cutoffs for *S. pneumoniae* in children are as low as the qLAMP detection limit, but rise to 1.62×10^5^ and 2.28×10^7^ copies/ml in adult patients without COPD. These results are in agreement with general clinical experiences and the literature. [13], [14].

### Epidemiologic Analysis Based on qLAMP Data

**Figure 4:**
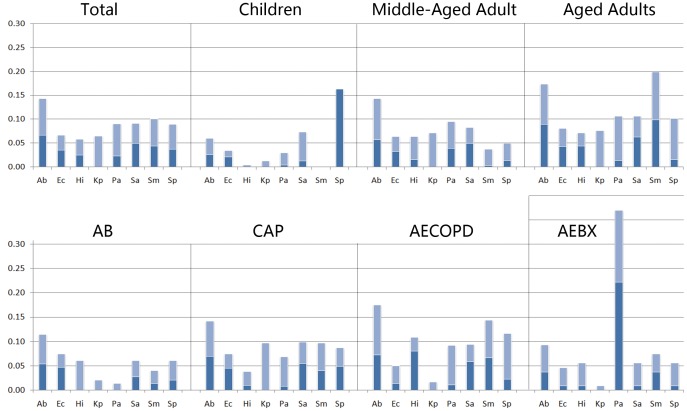
The percentage of bacteria identified as definite (dark blue) and possible (light blue) causative agents in LRTI patients (for the full bacterial names see the legend of **Figure 2** **).** The patients are classified as: children, ≤14 yr; middle-aged adult, >14 yr but <70 yr; aged, ≥70 yr; AB, acute bronchitis; CAP, community acquired pneumonia; AECOPD, acute exacerbation of COPD; and AEBX, acute exacerbation of bronchiectasis.

**Figure 5:**
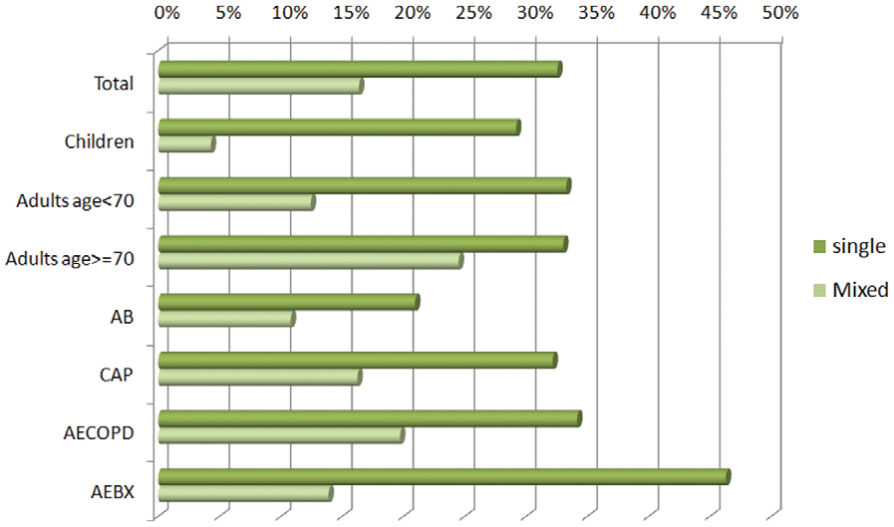
The qLAMP-based diagnostic rates in LRTI patients and different subgroups.

Based on titers quantified from qLAMP and cutoffs deduced from piecewise linear regression, we are able to identify the causative (definite or potential) pathogens for each patient subgroup (Figure 4). First, the major pathogens are *S. pneumoniae* (16.31%) in children and *P. aeruginosa* (37.04%) in AEBX (acute exacerbation of bronchiectasis) patients. Second, the fraction of infections from *A. baumannii, E. coli, S. aureus*, and *S. maltophilia* appears to increase together with age in all patients. Third, the diagnostic rates for single and mixed infections are 32.55% and 16.37%, respectively, making a total of 48.92%. Fourth, the diagnosis rates of total and mixed infections increase with age and the severity of infections (Figure 5). These results are also essentially in accordance with previous epidemiological studies. [15], [16].

## Discussion

### Quantitative LAMP is a Potential Diagnostic Tool for LRTI

DNA-based pathogen detection is becoming increasingly popular in recent years. More quantitative methods, such as real-time PCR and branch-DNA, provide adequate sensitivity for detecting even single copy of targeted DNA sequences and are thus capable of reaching nearly 100% specificity when used on the identification of standard pathogen strains. [17] LAMP represents a novel method in the same category that uses a single incubation temperature. It eliminates the need for expensive thermal cyclers, and has been applied in pathogen detection of infectious diseases, such as tuberculosis and malaria. [18], [19] It can be quantitative in that it measures turbidity or fluorescent signals from labeled DNA with similar sensitivity and specificity as real-time PCR. [20] Although quantitative LAMP (qLAMP) is more expensive than routine bacterial culture, the gross cost of the test is easily compensated by its ability to inform more accurate and timely etiological diagnosis. Therefore, qLAMP, or even other DNA-based methods, should have significant impacts on the etiological diagnosis for LRTI so long as these tests have acceptable sensitivity and specificity.

As all DNA-based methods do, qLAMP also has its potential drawbacks, such as the effect of sequence mutations on sensitivity and accuracy. In order to minimize this effect, we have deliberately selected the most characteristic and conservative genes as target sequences. However, it is still possible that clinical strains harbor mutations on the sequences where primers are designed. Fortunately, qLAMP is less affected by mutations than other methods, such as real-time PCR, for its long primers that cover over 160 bp in length. One or two mutations often do not make distinguishable effects on amplification. In our experience, only G/C→A mutations at the 3′ end of the most important primer–BIP or FIP–can lead to quantity undervaluation and lower sensitivity. However, our data show that the qLAMP method is much more sensitive than culture-based method. Therefore, the effect of mutations can not be a prevalent factor that influences the accuracy of qLAMP. Given the situation in our study, i.e., about 40% variations (changes at sites where primers are designed) in all sequenced strains and average 3.3 mutations in each mutant, the theoretical incidence of the effective mutation is only less than 0.5% in all strains. In addition, qLAMP has been successfully utilized in quantitative assays for many bacterial species and even viruses in clinical samples, [21], [22] while there has not been creditable evidence to suggest that qLAMP is very sensitive to sequence mutations of its targets in real cases.

### Could We Explain Discrepancies between DNA- and Culture-based Methods?

The clinical application of DNA-based methods in detecting bacteria has long been hampered by the discrepancies between their results and those from culture-based methods–most popular in clinical practice. The discrepancies essentially arise from their different detecting objects. For DNA-based methods, on the one hand, the targets for detection are representative sequences from the pathogens, alive or dead. Culture-based assays, on the other hand, detect all living bacterial cells grown in the medium. Certainly, there are more elements and factors to be considered when culture-based assays are to be performed, such as how to keep the cells alive and what selective media to use for visualizing the colonies. Therefore, statistic tools are of importance for dealing with the culture results. We indeed used logistic curve to describe the probability of a pathogen to be positive in culture and the titer of the pathogen panel to be detected in order to reduce ambiguity of the culture-based assays. The stochastic nature of the culture results can be both false negative, where inoculated bacteria become dead in the lengthy culturing process, and false positive, where bacteria are contaminants from the normal pharyngeal flora.

Sensitivity and specificity are two of the most important factors for evaluating new diagnostic tests. They are often used when a gold standard or composite reference with high sensitivity and specificity exits. [23] However, in our cases, culture test is the only other available reference, and it cannot be regarded as an ideal reference for determining the accuracy of qLAMP since it often produces stochastic results. In addition, we cannot apply any composite reference standard [24] due to unavailability of other reference tests aside from bacterial cultures, because blood cultures have even poorer positive rates, often less than 2% in Chinese populations [15] (including Hong Kong [25]). In our study, the well converged logistic regression demonstrates that qLAMP can detect target sequence rather stably and thus shows good reliability in bacterial titer quantification.

### Statistical Methods are of Essence in Determining Cut-offs Based on the Competitive Relationship among Bacterial Pathogens

It is always a challenge to differentiate causative pathogens from the pharyngeal flora that can be both “latent" and “contaminated". To make our DNA-based assay applicable to clinical settings, we have to filter out the influence of contaminations from the normal pharyngeal flora by setting rational cutoff values, or simply cutoffs, between causative and latent bacteria. To take the advantage of competitive relationships among pathogens, we assume that for a pathogen to become dominant it must go through a process from latency to dominance. Practically, we choose cutoffs for the different phases in the process, where the competitive relationship changes the most. Hundreds of microbial species inhabit in our pharynx, forming a changing yet complex microflora [26]. We made a few assumptions here. First, we assume that there is homeostasis in the entire microecosystem that lives through the host mucosal immunity, where all the microbial inhabitants form an interaction network to prevent a alien species from becoming dominant. [27]–[29] Second, we believe that under normal circumstances the pathogens are also controlled under seemingly normal conditions and do not have any more competitive or inhibitory effects over other microbial species. As a result, the pathogens are all in a latent or lag phase. Subsequently, when conditions are changed to be more suitable for one bacterial species over others, it may break the limit and propagate abnormally or start to inhibit the propagation of other species, and we say that the pathogen is in its log phase for fast growth. At the end, when the bacteria become dominant and destined to be pathogenic (dominant phase), the homeostasis of the microflora is totally destroyed and the inhibitory effect reaches its plateau. Therefore, third, we assume that cutoffs can be determined to separate latent and causative pathogens from breakpoints where pathogens escape from the control of host immunity and become fixed, showing characteristics of infection.

The breakpoint where a specific bacterial pathogen overcomes the host immunity control is determined by the stability of its niche in pharynx. [30] Although both the components and their abundances in the microflora vary among individuals, indicators, such as the diversity of the whole microbiome, can be studied and described in molecular terms within human populations. [31], [32] Therefore, we can assume that patients from the same subgroup should share pharyngeal microecosystems or at least the properties of their flora and thus share similar breakpoints as well. This assumption is supported by our observations. For instance, when the regression curves do not converge well, the breakpoints are often heterogeneous over stratified populations or subgroups. In other words, the rational stratification of populations or patients should improve convergence and the shared cutoffs among similar populations added a footnote to this notion.

### The Cutoffs Derived from Piecewise Linear Regress has Clinical Implications

The cutoffs derived from piecewise linear regression provide information for clinical treatments. For instance, if a titer determined based on qLAMP is above the upper cutoff, the bacterium is considered to be the causative pathogen of the infection, and relevant antibiotics should be prescribed. If the titer is below the lower cutoff, the bacterium is considered to be normal colonized status in the host and antibiotic treatment is deemed unnecessary. If the titer falls between the two cutoffs, the bacterium is possible to be involved in causing infection and should be cared for by selecting an appropriate antibiotic treatment.

Although determining cutoffs for the differentiation of causative pathogens from normal flora in sputum samples is highly desirable in the etiological diagnosis of LRTI, it cannot be easily obtained based on routine statistical methods due to the lack of sputum samples from a legitimate control group. In our study, this problem is overcome by using statistic tools based on the competitive nature of bacterial pathogens and their growth characteristics when becoming pathogenic. It can be applied in cases that meet the following conditions. First, quantitative data for multiple pathogens are available. Second, competitive relationship among the panel of pathogens is unbiased. Third, the trial size is large enough to resist stochastic factors. Therefore, cutoffs for pathogens can be deduced, regardless if the pathogens can be cultured or not, based on the statistic methods as long as they exhibit both latent and pathogenic phases in the host pharyngeal flora.

### Cautions

Some bacterial species in our study show somewhat higher rates as causative agents than expected. The reasons may fall into either or both of the following categories. First, the target genes selected or primers designed are not conserved enough or optimized enough to have satisfactory sensitivity and specificity to be accurately quantified. Second, the subjects in our study are all in-patients and their symptoms are somewhat more severe than what often observed in common CAP and AECOPD patients. It should also be mentioned that the number of patients in our study is rather limited when the data are stratified into subgroups. Therefore, we noticed heterogeneity in breakpoints in the same patient subgroup, which affects the accuracy in deducing cutoffs. However, this last caution can be eliminated by increasing the cohort size, and implementing better experimental designs in future studies.

### Conclusion

In conclusion, we demonstrated that qLAMP assay (and perhaps other quantitative DNA-based methods) is a reliable method for the quantification of pathogens in sputum samples, especially when statistical analyses based on competitive relationships among bacteria are appropriately applied to determine thresholds where pathogens can overcome them and become pathogenic afterwards. These thresholds can also serve as cutoffs to filter out influence of contaminants from the pharyngeal flora to make qLAMP as quantitative methods practical in clinical etiologic diagnosis. We believe that quantitative DNA-based assays will make important contributions in the future to the clinical identification of pathogens, including bacteria, non-classical pathogens, and fungi, especially in the present time when pathogens are more complex than before, and some of them are not only presently unknown but also not easily identified based on the routine culture methods.

## Supporting Information

Figure S1 **Patient distribution in different provinces of China.** The provinces where we recruited patients are shown in grey and the numbers of cases are heighted in red. The names of Provinces or Municipalities from are listed in order as follows (north to south): Inner Mongolia Autonomous Region (116 cases), Liaoning Province (58 cases), Beijing (383 cases), Gansu Province (107 cases), Henan Province (47), Jiangsu Province and Shanghai (91), Hubei Province (56 cases), Guizhou Province (39 cases), Hunan Province (145 cases), Jiangxi Province (126 cases), Fujian Province (147 cases), Guangxi Zhuang Autonomous Region (11 cases), and Guangdong Province (93 cases).
(DOCX)

Figure S2 **Diagram of the strategy for the LAMP primer design.**
(DOCX)

Figure S3 **Logistic regression curves.** Solid circles indicate patients; they are placed at the top of the chart when being test as positive and at the bottom of the chart when being tested as negative in the culture assays. The height and width of the bars display the frequency and the number of patients being tested positive in cultures, respectively. The titters are divided in a natural logarithmic scale.
(DOCX)

Figure S4 **Piecewise linear regression data.** Open circles indicate patients; they are placed at the top of the chart when being PC and at the bottom of the chart when NOT being PC. The titters are divided in a common logarithmic scale.
(DOCX)

Table S1 **Primers originated from the specific gene for bacterial species identification.**
(DOCX)

Table S2 **Primer sequence list for each bacterial species.**
(DOCX)

Table S3 **The list of target and reference bacterial species.**
(DOCX)

Table S4 **The list of hospitals and recruited cases.**
(DOCX)

Table S5 **Demographic characteristics and LITR diagnoses of qualified patients.**
(DOCX)

Table S6 **The cut-offs in different subgroups.**
(DOCX)

Checklist S1 **STARD Checklist.**
(DOC)

Methods S1 (DOCX)

Protocol S1 **Trial Protocol.**
(DOC)
