# Supplementary material for: Etiologic Diagnosis of Lower Respiratory Tract Bacterial Infections Using Sputum Samples and Quantitative Loop-Mediated Isothermal Amplification
Source: PLoS One. 2012 Jun 14;7(6):e38743. doi: 10.1371/journal.pone.0038743 (PMC3375278; doi:10.1371/journal.pone.0038743)
Supplement: Figure S1 — Patient distribution in different provinces of China. The provinces where we recruited patients are shown in grey and the numbers of cases are heighted in red. The names of Provinces or Municipalities from are listed in order as follows (north to south): Inner Mongolia Autonomous Region (116 cases), Liaoning Province (58 cases), Beijing (383 cases), Gansu Province (107 cases), Henan Province (47), Jiangsu Province and Shanghai (91), Hubei Province (56 cases), Guizhou Province (39 cases), Hunan Province (145 cases), Jiangxi Province (126 cases), Fujian Province (147 cases), Guangxi Zhuang Autonomous Region (11 cases), and Guangdong Province (93 cases). (DOCX) [file pone.0038743.s001.docx]

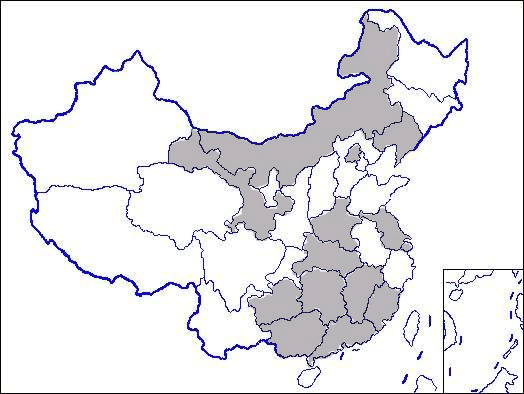


39

11

93

145

147

126

56

91

47

107

58

383

116

**Figure S1. Patients distribution and the recruited number in different provinces in China.**

The province from which we finally recruited patients is shown in grey, with the patients number red labeled within it. The names of Provinces or Municipalities from North to South are listed in order as followings: Inner Mongolia Autonomous Region (116 cases), Liaoning Province (58 cases), Beijing (383 cases), Gansu Province (107 cases), Henan Province (47), Jiangsu Province and Shanghai (91), Hubei Province (56 cases), Guizhou Province (39 cases), Hunan Province (145 cases), Jiangxi Province (126 cases), Fujian Province (147 cases), Guangxi Zhuang Autonomous Region (11 cases), Guangdong Province (93 cases).
