# Supplementary figures and images for: Etiologic Diagnosis of Lower Respiratory Tract Bacterial Infections Using Sputum Samples and Quantitative Loop-Mediated Isothermal Amplification
Source: PLoS One. 2012 Jun 14;7(6):e38743. doi: 10.1371/journal.pone.0038743 (PMC3375278; doi:10.1371/journal.pone.0038743)

**
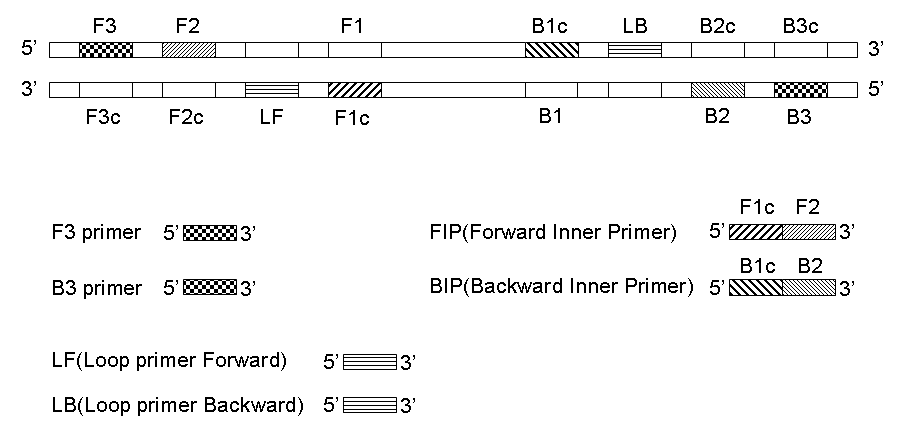
Figure S2. Diagram of the contrivable strategies of LAMP primer location.**

Supplement: Figure S2 — Diagram of the strategy for the LAMP primer design. (DOCX) [file pone.0038743.s002.docx]
