# Supplementary material for: Etiologic Diagnosis of Lower Respiratory Tract Bacterial Infections Using Sputum Samples and Quantitative Loop-Mediated Isothermal Amplification
Source: PLoS One. 2012 Jun 14;7(6):e38743. doi: 10.1371/journal.pone.0038743 (PMC3375278; doi:10.1371/journal.pone.0038743)
Supplement: Figure S3 — Logistic regression curves. Solid circles indicate patients; they are placed at the top of the chart when being test as positive and at the bottom of the chart when being tested as negative in the culture assays. The height and width of the bars display the frequency and the number of patients being tested positive in cultures, respectively. The titters are divided in a natural logarithmic scale. (DOCX) [file pone.0038743.s003.docx]

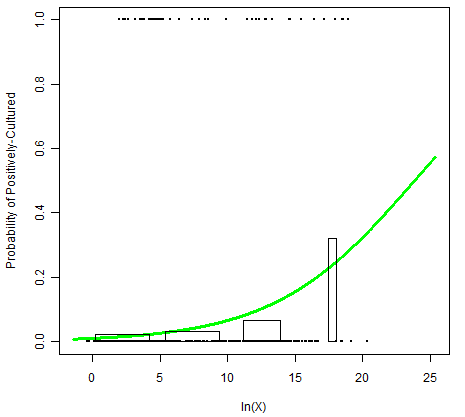


***A. baumannii E. coli*  *H. influenza* *K. pneumoniae***

***P. aeruginosa* *S. aureus* *S. maltophilia* *S. pneumonia***

**Figure S3. Logistic regression curves.**

Solid circles indicate patients; they are placed at the top of the chart when being test as positive and at the bottom of the chart when being tested as negative in the culture assays. The height and width of the bars display the frequency and the number of patients being tested positive in cultures, respectively. The titters are divided in a natural logarithmic scale.
