# Supplementary material for: Etiologic Diagnosis of Lower Respiratory Tract Bacterial Infections Using Sputum Samples and Quantitative Loop-Mediated Isothermal Amplification
Source: PLoS One. 2012 Jun 14;7(6):e38743. doi: 10.1371/journal.pone.0038743 (PMC3375278; doi:10.1371/journal.pone.0038743)
Supplement: Figure S4 — Piecewise linear regression data. Open circles indicate patients; they are placed at the top of the chart when being PC and at the bottom of the chart when NOT being PC. The titters are divided in a common logarithmic scale. (DOCX) [file pone.0038743.s004.docx]

Probability of being PC


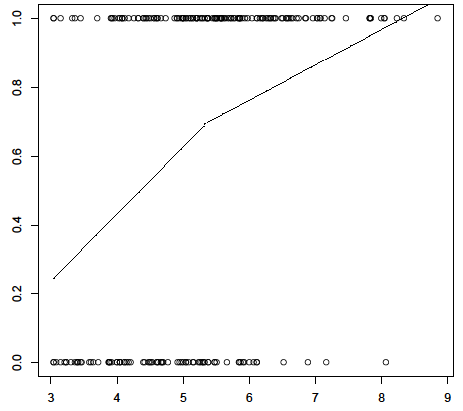

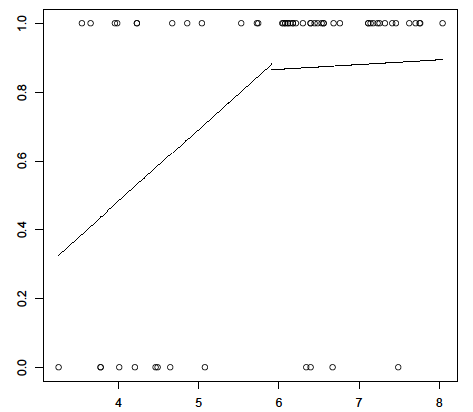

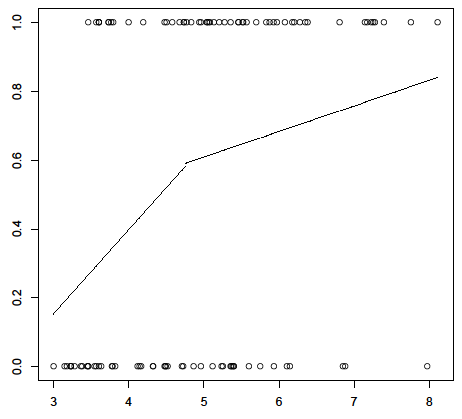

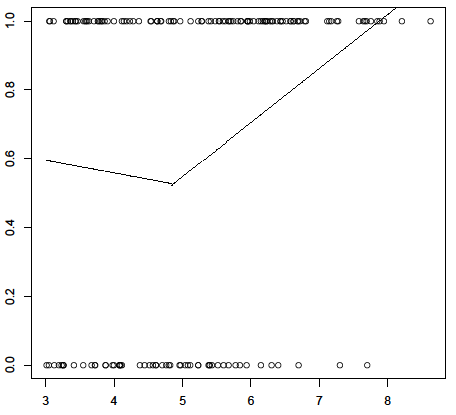

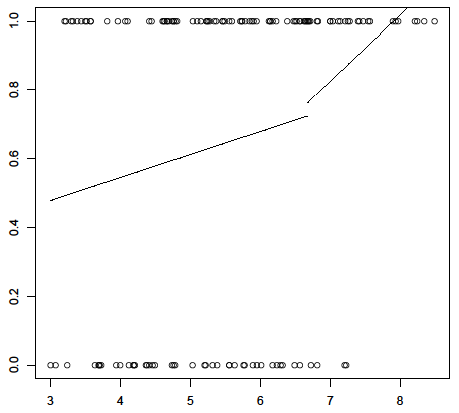


***A. baumannii E. coli* *H. influenzae* in COPD patients *H. influenza* in non-COPD patients *K. pneumonia* in CAP patients**


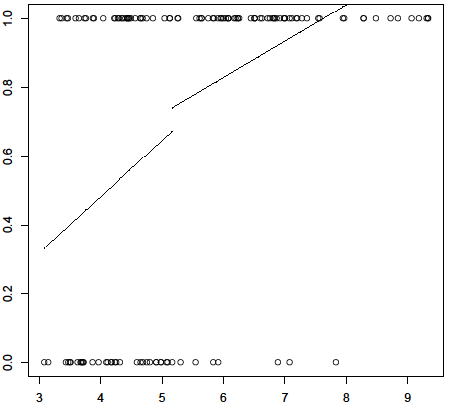

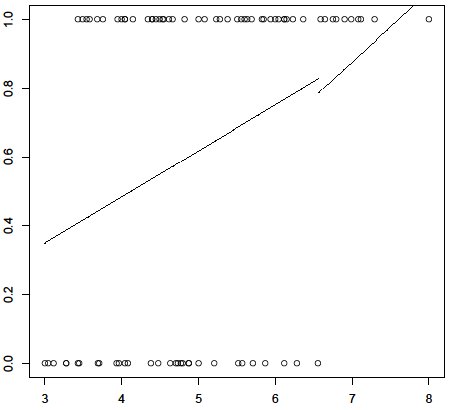

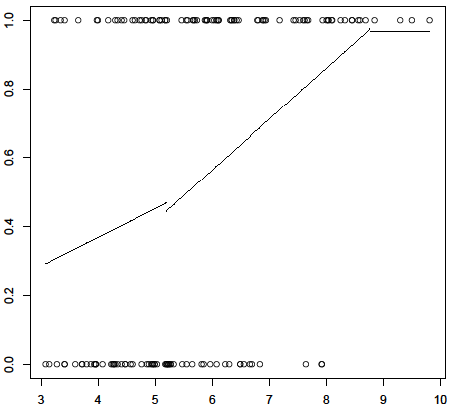

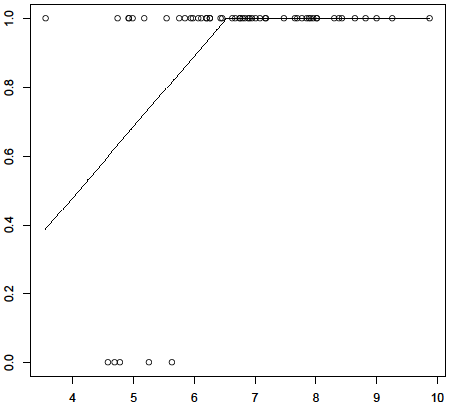

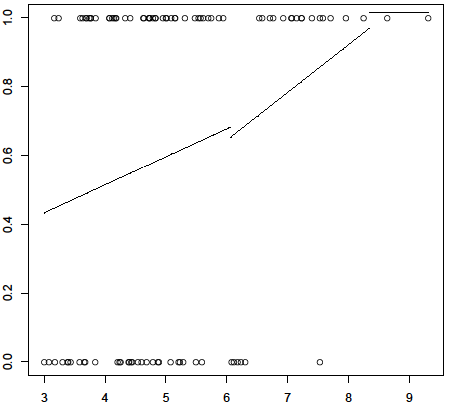


Probability of being PC

***K. Pneumoniae* in non-CAP patients *P. Aeruginosa* in BX patients *P. Aeruginosa* in non-BX patients *S. maltophilia* in Aged patients *S. maltophilia* in non-Aged patients**

Probability of being PC


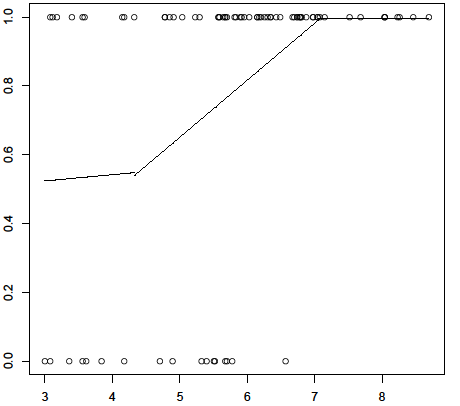

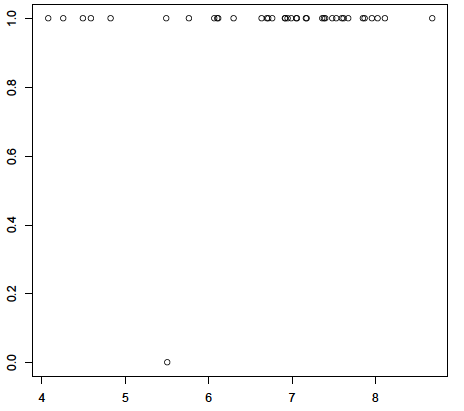

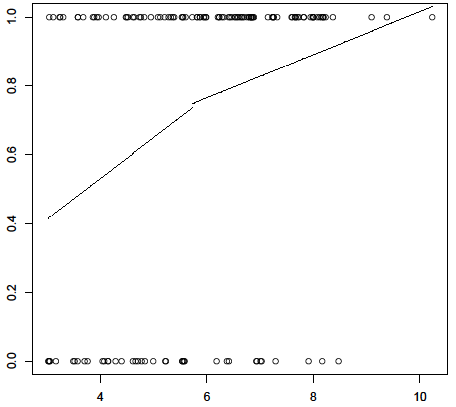

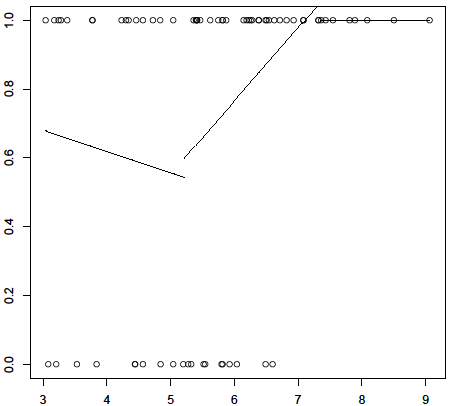


***S. aureus*  *S. pneumoniae* in Children patients *S. pneumoniae*--- adult COPD patients *S. pneumoniae*---adult non-COPD patients**

**Figure S4. Piecewise linear regression data**.

Solid circles indicate patients; they are placed at the top of the chart when being test as PC and at the bottom of the chart when being tested as not PC. The titters are divided in a common logarithmic scale.
