# Supplementary material for: Etiologic Diagnosis of Lower Respiratory Tract Bacterial Infections Using Sputum Samples and Quantitative Loop-Mediated Isothermal Amplification
Source: PLoS One. 2012 Jun 14;7(6):e38743. doi: 10.1371/journal.pone.0038743 (PMC3375278; doi:10.1371/journal.pone.0038743)
Supplement: Table S1 — Primers originated from the specific gene for bacterial species identification. (DOCX) [file pone.0038743.s005.docx]

**Table S1. Primers originated from the specific gene for bacterial spiece identification.**

| **Species** | **Gene** | **GenBank accession No.** |
| --- | --- | --- |
| *Streptococcus pneumoniae* | *lyt*A | AJ243399 |
| *Staphylococcus aureus* | *fem*A | BA000033 |
| *Escherichia coli* | *pho*A | U00096 |
| *Klebsiella pneumoniae* | KPN_04473 | CP000647 |
| *Pseudomonas aeruginosa* | *opr*I | M25761 |
| *Acinetobacter baumannii* | *ade*S | CP000521 |
| *Stenotrophomonas maltophilia* | *stm*Pr | AY253983 |
| *Haemophilus influenzae* | *omp*P6 | CP000671 |
