# Supplementary material for: Etiologic Diagnosis of Lower Respiratory Tract Bacterial Infections Using Sputum Samples and Quantitative Loop-Mediated Isothermal Amplification
Source: PLoS One. 2012 Jun 14;7(6):e38743. doi: 10.1371/journal.pone.0038743 (PMC3375278; doi:10.1371/journal.pone.0038743)
Supplement: Table S2 — Primer sequence list for each bacterial species. (DOCX) [file pone.0038743.s006.docx]

**Table S2 Primer sequence lists for different bacterial species**

| **Species** | **Primer** | **Sequence** |
| --- | --- | --- |
| *S. pneumoniae* | F3 | CTGGAGGAAGCACACAGA |
|  | B3 | GTCTGGTTTGAGGTAGTACC |
|  | FIP | CACCTTCTTCGTTGAAATAGTACCA-CTGGTTCGACAACTCAGG |
|  | BIP | GACAGGCTGGGTCAAGTACAA-TGGATAAAGGCATTTGATACC |
|  | LF | AGCGATTTTCTTCCAGCC |
|  | LB | CTTAGACGCTAAAGAAGGCG |
| *S. aureus* | F3 | TGAATCATGATGGCGAGAT |
|  | B3 | CGTGTTTCTTTTTCTAAGTCCA |
|  | FIP | ATGGAATCCAGTATGTTCAAATCCT-AGGTAATGCTGGTAATGATTGG |
|  | BIP | AAGGATTTGATCCTGTGCTACAAA-TTTAATGATGTCATCTGCTGTT |
|  | LF | AAGTTACTCATTTTATCAAAGA |
|  | LB | TTCGTTATCACTCAGTGTTAGA |
| *E. coli* | F3 | GCGCGTGGTTATCAGTTGG |
|  | B3 | CCAGGGTTGGTACACTGTC |
|  | FIP | CGTCAGCAAACAGGCCAAGCA-TCACTGAATTCGGTGACGGA |
|  | BIP | AGGACCGAAAGCAACGTACCAC-GCGGATTAGGCGTACAGG |
|  | LF | GGGGTTTTTGCTGATTCGCT |
|  | LB | TATCGATAAGCCCGCAGTCA |
| *K. pneumoniae* | F3 | CGCCACTATCGACAGTCAG |
|  | B3 | TGCAGACCGGTAAAACTCAA |
|  | FIP | ATCGCTGTGGCTATAGGTGCTG-AGCCTGGGCTGAATCTGG |
|  | BIP | TCTTGCCCGCGATATTCACACC-CGAAAATGCCGGAAGAGGTA |
|  | LF | GCAGGCGCCATGGTC |
|  | LB | AGCCAGCTGGTGGTCG |
| *P. aeruginosa* | F3 | CTGGATATTTTTTGAACAAACGA |
|  | B3 | GTTCATCGTGTTCCCCTTA |
|  | FIP | TAAACTGACCAAGCGCAAGC-CCAACTACTGCTAAAGTCGG |
|  | BIP | ACAAGTAATGGGTAGTATGTAGCCG-GGACATTTCCATAACAGCAATC |
|  | LF | AGCAACTTTTTTTTTAGTCCCC |
|  | LB | GCTAATTTCCCCGGCTG |
| *A. baumannii* | F3 | TGTGCCAATTAACTTCTTAGC |
|  | B3 | CTTGTAATATCGTTATAGGCGTT |
|  | FIP | AGTGAATTCGGTTATCGTAAGCTC-GAAGCAGCAAAAAAAATTAGTCAC |
|  | BIP | AATGATATGGCTCAAAAGCTAGAGG-CTTAACTCATGTGCGATGG |
|  | LF | TAGCAGAGAGGTCGCC |
|  | LB | CCGTTAAAAATGCGCAGG |
| *S. maltophilia* | F3 | ATGCCGACCTGTACGT |
|  | B3 | CCCATGGAGAGGGTCTTG |
|  | FIP | GTCTCGCTGTTGCCGCTCA-CAGTGCCCCGACCGATA |
|  | BIP | GTGGTATGTGCGGGTGAAGG-AGTACTGGGCGTTGAGG |
|  | LF | TACGGACGGCAGGTGTA |
|  | LB | TACAGCACCTTCTCGGG |
| *H. influenzae* | F3 | GCAGATGCAGTTAAAGGTT |
|  | B3 | GCTAATTGGTTAAATTACAAACGA |
|  | FIP | ACCTAATACTGCAGGTTTTTCTTCA-GGTAAAGGTGTTGATGCTGG |
|  | BIP | GAAGCTGCATATTCTAAAAACCGTC-AAAAATGGATCCTGTTTTTCAAGT |
|  | LF | CCGTAAGATACTGTGCCTAATT |
|  | LB | GCAGTGTTAGCGTACTAATTCT |
