# Supplementary material for: Etiologic Diagnosis of Lower Respiratory Tract Bacterial Infections Using Sputum Samples and Quantitative Loop-Mediated Isothermal Amplification
Source: PLoS One. 2012 Jun 14;7(6):e38743. doi: 10.1371/journal.pone.0038743 (PMC3375278; doi:10.1371/journal.pone.0038743)
Supplement: Table S3 — The list of target and reference bacterial species. (DOCX) [file pone.0038743.s007.docx]

**Table S3. The lists of target and reference bacterial species**

| **Species** | **Strain** | **Molecular Size (Mbp)** |
| --- | --- | --- |
| **8 pneumonia-related species** |  |  |
| *Streptococcus pneumoniae* | CMCC(B)* 31206 CMCC(B) 31210 | 2.1 |
| *Staphylococcus aureus* | CMCC(B) 26112 CMCC(B) 26075 | 2.8 |
| *Escherichia coli* | CMCC(B) 44113 CMCC(B) 44820 | 5.2 |
| *Klebsiella pneumoniae* | CMCC(B) 46117 CMCC(B) 46114 | 4.9 |
| *Pseudomonas aeruginosa* | CMCC(B) 10115 CMCC(B) 10120 | 6.4 |
| *Acinetobacter baumannii* | ATCC^$^ 19606  Clinical isolate | 4.0 |
| *Stenotrophomonas maltophilia* | ATCC 13637  Clinical isolate | 4.5 |
| *Haemophilus influenzae* | ATCC 49247  ATCC 49766 | 1.8 |
| **19 other species** |  |  |
| *Enterobacter cloacae* | CGMCC^†^ 1.57 CGMCC 1.58 | 4.6 |
| *Enterobacter aerogenes* | CMCC(B) 45103 CGMCC 1.2021 | 4.6 |
| *Citrobacter freundii* | CGMCC 1.1732 | 4.7 |
| *Citrobacter sp.* | CMCC(B) 48032 | 4.7 |
| *Acinetobacter calcoaceticus* | CGMCC 1.2004 | 4.0 |
| *Proteus vulgaris* | CGMCC 1.1527 | 4.0 |
| *Proteus mirabilis* | CMCC(B) 49106 | 4.0 |
| *Staphylococcus epidermidis* | Clinical isolate | 2.6 |
| *Streptococcus pyogenes* | CMCC(B) 32008 | 1.9 |
| *Streptococcus agalactiae* | CVCC^‡^ 586 | 2.2 |
| *Lactococcus lactis* | CVCC 590 | 2.5 |
| *Streptococcus salivarius* | CVCC 5911 | 2.0 |
| *Enterococcus faecalis* | CMCC(B) 32223 | 3.4 |
| *Corynebacterium diphtheriae* | CMCC(B) 38201 | 2.5 |
| *Corynebacterium pseudodiphtheriticum* | CMCC(B) 38203 | 2.5 |
| *Corynebacterium diphtheriae* | *CMCC(B) 38003* | *2.5* |
| *Legionella pneumophila* | ATCC 33152 | 3.4 |
| *Mycoplasma pneumoniae* | ATCC 15531 | 0.8 |
| *Chlamydophila pneumoniae* | ATCC VR-2282 | 1.2 |

$ ATCC, American Type Culture Collection

* CMCC(B), National Center for Medical Culture Collection (Bacteria), Beijing, China

† CGMCC, China General Microbiological Culture Collection Center, Beijing, China

‡ CVCC, China Veterinary Culture Collection Center, Beijing, China
