# Supplementary material for: Etiologic Diagnosis of Lower Respiratory Tract Bacterial Infections Using Sputum Samples and Quantitative Loop-Mediated Isothermal Amplification
Source: PLoS One. 2012 Jun 14;7(6):e38743. doi: 10.1371/journal.pone.0038743 (PMC3375278; doi:10.1371/journal.pone.0038743)
Supplement: Table S4 — The list of hospitals and recruited cases. (DOCX) [file pone.0038743.s008.docx]

**Table 4. The list of attending hospitals, recruited patient’s number in each hospital and hospital’s location in China.**

|  | **Hospital** | **Number of Cases** |
| --- | --- | --- |
| **North China** | Peking University Space Central Hospital | 9 |
|  | Third Affiliated Hospital of the Inner Mongolia Medical College | 116 |
|  | Peking University Third Hospital | 29 |
|  | First Affiliated Hospital of Tsinghua University University | 43 |
|  | Beijing Children's Hospital affiliated of Capital Medical University | 235 |
|  | No.263 Clinical Section of the Military General Hospital of Beijing | 67 |
| **Northeast China** | First Affiliated Hospital of China Medical University | 58 |
| **Northwest China** | Lanzhou Pulmonary Hospital | 11 |
|  | First Hospital of Lan Zhou University | 96 |
| **East China** | Fujian Provincial Hospital | 147 |
|  | First Affiliated Hospital of Nanchang University | 126 |
|  | Lianyungang First People's Hospital affiliated of Xuzhou Medical College | 91 |
|  | Ruijin Hospital affiliated to Shanghai Jiaotong University | 114 |
| **Central South China** | First Affiliated Hospital of Guangxi Medical University | 11 |
|  | People’s hospital of Wuhan University | 56 |
|  | Third Affiliated Hospital of Guangzhou Medical College | 93 |
|  | Xiangya Hospital of Central South University | 145 |
|  | Henan Province People’ Hospital | 47 |
| **Southwest China** | Affiliated Hospital of Guiyang Medical College | 39 |
| **Total** |  | 1533 |
