# Supplementary material for: Etiologic Diagnosis of Lower Respiratory Tract Bacterial Infections Using Sputum Samples and Quantitative Loop-Mediated Isothermal Amplification
Source: PLoS One. 2012 Jun 14;7(6):e38743. doi: 10.1371/journal.pone.0038743 (PMC3375278; doi:10.1371/journal.pone.0038743)
Supplement: Table S5 — Demographic characteristics and LITR diagnoses of qualified patients. (DOCX) [file pone.0038743.s009.docx]

**Table S5. Demographic characteristics and LITR diagnoses.**

|  | **AB** | **CAP** | **AECOPD** | **AEBX** | **Children** | **Adult (<70yr)** | **Adults (>=70yr)** | **Total** |
| --- | --- | --- | --- | --- | --- | --- | --- | --- |
| **Number of patients** | 148 | 917 | 360 | 108 | 233 | 643 | 657 | 1533 |
| **Median age** | 61.5 | 60 | 74 | 60 | 5 | 53 | 77 | 63.43 |
| **Age range** | 1-94 | 0-95 | 33-93 | 3-87 | 0-14 | 15-70 | 70-95 | 0-95 |
| **Female%** | 36.5% | 32.1% | 23.3% | 44.4% | 36.5% | 34.2% | 26.5% | 31.3% |

Note: AB—Acute bronchitis; CAP—Community-acquired pneumonia; AECOPD—Acute exacerbation of COPD; AEBX— Acute exacerbation of bronchiectasis; Children—Patients with age =<14yr; Adult (<70yr)—Patients with age > 14yr and < 70yr; Adults (>=70yr) —Patients with age > = 70yr
