# Supplementary material for: Etiologic Diagnosis of Lower Respiratory Tract Bacterial Infections Using Sputum Samples and Quantitative Loop-Mediated Isothermal Amplification
Source: PLoS One. 2012 Jun 14;7(6):e38743. doi: 10.1371/journal.pone.0038743 (PMC3375278; doi:10.1371/journal.pone.0038743)
Supplement: Table S6 — The cut-offs in different subgroups. (DOCX) [file pone.0038743.s010.docx]

**Table S6 The decreases of cut-offs in susceptive subgroups**

| **non-specific** | **Children** | **Aged patients** | **AECOPD** | **AEBX** | **CAP** |
| --- | --- | --- | --- | --- | --- |
| *A. baumannii* |  |  |  |  |  |
| *H. influenzae* |  |  | ↓↓ |  |  |
| *K. pneumoniae* |  |  |  |  | ↓↓ |
| *P. aeruginosa* |  |  |  | ↓↓ |  |
| *S. maltophilia* |  | ↓↓ |  |  |  |
| *S. Pneumoniae* | ↓↓ |  | ↓ |  |  |
