# Supplementary material for: Etiologic Diagnosis of Lower Respiratory Tract Bacterial Infections Using Sputum Samples and Quantitative Loop-Mediated Isothermal Amplification
Source: PLoS One. 2012 Jun 14;7(6):e38743. doi: 10.1371/journal.pone.0038743 (PMC3375278; doi:10.1371/journal.pone.0038743)
Supplement: Methods S1 — (DOCX) [file pone.0038743.s012.docx]

**Methods**

**qLAMP**

The template for LAMP was prepared by a three-step protocol: (1) Liquefaction. 500 µL sputum from each sample was mixed with 500 µL NaOH (10%). The mixture was incubated at room temperature for 30 min and then centrifuged at 10000×g for 5 min. (2) Washing. The pellets were resuspended in 1 mL saline solution and centrifuged at 10000×g for 5 min. (3) Cell lysis (using the Universal Kit for Bacterial DNA Extraction, Capitalbio Corporation, P. R. China). The pellets were resuspended in 100 µL 1×TE buffer (10 mM Tris-HCl, 1 mM EDTA, pH 8.0) and transfered to a new tube containing 50 mg glass bead mixture(150-212, 710-1180, Sigma-Aldrich Co., MO, USA). Each sample was vortexed for 10 min, incubated at 95°C for 10 min and centrifuged at 2000×g for 1 min. The supernatant was transferred to a sterile tube and stored at -20°C until LAMP testing.

The LAMP primers were designed based on the strategy previously described by Notomi ^[1, 2]^ and synthesized commercially. That is a set of six primers, consisting of two outer (F3 and B3) and two inner (FIP and BIP) primers, and two loop (LF and LB) primers, capable of recognizing eight distinct regions on each target sequence was designed for each target species. FIP consists of a complementary sequence of F1 (F1c) and a sense sequence of F2. Similarly, BIP consists of a complementary sequence of B1 (B1c) and a sense sequence of B2, as shown in eFigure 3

Quantitative LAMP assay was performed by real-time fluorescence as before^[3]^, and briefly in 25μl reaction mixture containing 1.6μM each of FIP and BIP, 0.2μM each of F3 and B3, 0.4μM each of LF and LB, 8U of the Bst DNA polymerase large fragment (New England Biolabs Inc., Beverly, Mass., USA), 0.4mM dNTP, 0.1mM dUTP, 0.8M betaine, 6mM MgSO_4_, 0.5mg/ml BSA, 0.6 × EvaGreen (Biotium, Inc., CA, USA), 0.1U/ml Uracil-DNA Glycosylase (Fermentas Inc., MD, USA), 20mM Tris-HCl (pH 8.8 at 250C), 10mM KCI, 10 mM (NH_4_) _2_SO_4_, 0.1%Triton X-100, and 2μl template DNA or PCR grade H_2_O as negative control. All amplifications were performed with a RT-Cycler Real-time Fluorescence Quantitative PCR Instrument (CapitalBio Corporation, Beijing, China ) as incubated at 65^0^C for 45 min and then heated at 80^0^C for 5 min to terminate the reaction. Amplification data were analyzed by software applied with the RT-Cycler and calculated the time to reach thresholds (Tt)

Quantified DNA of the 8 tested bacteria, which was extracted and purified by QIAamp according to the manufacturer’s instructions, was serially diluted as positive control. Concentration of copy-number of genomic equivalents was calculated based on its genome size to plot standard curve. Then the equivalent copy numbers of the template DNA were determined based on the standard curves generated from quantified control DNA.

The sensitivity of each set of primers was determined by serially diluted each targeted DNA template as the consistently detectable minimal content (copies/μl DNA template), then traced to the titer of bacteria in sputum by the following equation:

Titer in sputum [copies/ml] = copies/μl (content in DNA template) × 100μl (template volume) / 0.5 ml (sputum volume)

The specificity of each set of primers were evaluated by quantified bacteria DNA of 27 species (8 targeted and 19 reference species, listed in Supplement Table 4) obtained as mentioned above, to exclude primer set show any cross reaction with other species DNA.

Serially diluted each targeted DNA templates were tested at least 8 times at different time and on different machines. The ranges of error were calculated to evaluate the reproducibility of our LAMP system.

**Logistic model**

Because the detection limit of LAMP in our study was 10^3^copies/ml, there must be some cases who carried bacteria with titer lower than 10^3^copies/ml and were not detected by LAMP. These cases still had small probabilities to be positively-cultured, and should not be neglected in regression calculation. Because their accurate titer values were unknown, we use a randomizing approach to assign their titer for regression, (i.e. to consider their titer was normally distributed with 99% within the interval (0, 10^3^). Thereby, we used all the 1533 cases in regression calculation for each species.

In the regression, we extended the linear model ‘Y = *β_0_* + *β_1_*X + *e’* to nonlinear modal ‘Y =*β_0_* + *β_1_* ln (X) + *e’*, which was supported by Wald-test.

**References**

1. Notomi T, Okayama H, Masubuchi H, Yonekawa T, Watanabe K, Amino N, Hase T. Loop- mediated isothermal amplification of DNA. Nucleic Acids Res. 2000 Jun 15;28(12):E63

2. Nagamine K, Hase T, Notomi T. Accelerated reaction by loop-mediated isothermal amplification using loop primers. Mol Cell Probes. 2002 Jun;16(3):223-9.

3. Aoi Y, Hosogai M, Tsuneda S. Real-time quantitative LAMP (loop-mediated isothermal amplification of DNA) as a simple method for monitoring ammonia-oxidizing bacteria. J Biotechnol. 2006 Oct 1;125(4):484-91. Epub 2006 Jun 21.
