# Supplementary material for: Etiologic Diagnosis of Lower Respiratory Tract Bacterial Infections Using Sputum Samples and Quantitative Loop-Mediated Isothermal Amplification
Source: PLoS One. 2012 Jun 14;7(6):e38743. doi: 10.1371/journal.pone.0038743 (PMC3375278; doi:10.1371/journal.pone.0038743)
Supplement: Protocol S1 — Trial Protocol. (DOC) [file pone.0038743.s013.doc]

**Clinical Research Protocol**

**ProspecTIve, multi-center, clinical study of quantitative LAMP vs routine culture / Serological test for etiologic diagnosis in LRTI patients**

| **PI:** | Zhancheng Gao M.D., Ph.D. |
| --- | --- |
| **Protocol No.** | 00001384 |
| **Regulary Sponsor:** | Department of of Respiratory and Critical Care Medicine, Peking University People's Hospital |
| **Funding Sponsor:** | Grant from the Ministry of Science and Technology of P.R.China. (National High-Tech R&D program: 2006AA02Z4A9) |
| **Diagnostic test:** | Quantitative LAMP (Loop-mediated isothermal AMPlification)  Routine bacterial culture/serological test |
| **Contact:** | Yu Kang  Tel: 010-88324279  Fax: 010-68318386  E-mail: cangubwd@sina.com |

**Initial version:** 12th April 2007

**Table of Contents**

Study Summary [1](#__RefHeading___Toc284347461)

1 Introduction [2](#__RefHeading___Toc284347462)

1.1 Background [2](#__RefHeading___Toc284347463)

1.2 Index test [2](#__RefHeading___Toc284347464)

1.3 Preclinical Data [2](#__RefHeading___Toc284347465)

2 Study Objectives [3](#__RefHeading___Toc284347466)

3 Subject Selection [3](#__RefHeading___Toc284347467)

3.1 Inclusion Criteria [3](#__RefHeading___Toc284347468)

3.2 Exclusion Criteria [3](#__RefHeading___Toc284347469)

3.3 Subject Recruitment and Screening [3](#__RefHeading___Toc284347470)

4 Study Design and Procedures [4](#__RefHeading___Toc284347471)

5 Statistical Plan [5](#__RefHeading___Toc284347472)

5.1 Sample Size [5](#__RefHeading___Toc284347473)

5.2 Statistical Methods [5](#__RefHeading___Toc284347474)

5.3 Subject Population(s) for Analysis [5](#__RefHeading___Toc284347475)

6 Safety and Adverse Events [5](#__RefHeading___Toc284347476)

7 Data Handling and Record Keeping [5](#__RefHeading___Toc284347477)

7.1 Confidentiality [5](#__RefHeading___Toc284347478)

7.2 Source Documents [5](#__RefHeading___Toc284347479)

7.3 Case Report Forms [5](#__RefHeading___Toc284347480)

7.4 Records Retention [6](#__RefHeading___Toc284347481)

8 Study Monitoring, Auditing, and Inspecting [6](#__RefHeading___Toc284347482)

8.1 Study Monitoring Plan [6](#__RefHeading___Toc284347483)

8.2 Auditing and Inspecting [6](#__RefHeading___Toc284347484)

9 Ethical Considerations [6](#__RefHeading___Toc284347485)

10 Study Finances [7](#__RefHeading___Toc284347486)

10.1 Funding Source [7](#__RefHeading___Toc284347487)

10.2 Conflict of Interest [7](#__RefHeading___Toc284347488)

11 Publication Plan [7](#__RefHeading___Toc284347489)

12 References [7](#__RefHeading___Toc284347490)

13 Attachments (have not translated to English) [7](#__RefHeading___Toc284347491)

**List of Abbreviations**

LRTI Lower Respiratory Tract Bacterial Infection

LAMP Loop-mediated isothermal AMPlification

qLAMP quantitative Loop-mediated isothermal AMPlification

# Study Summary

| Title | Prospective, multi-center, clinical study of quantitative LAMP vs. routine culture/serological test for etiologic diagnosis in LRTI patients |
| --- | --- |
| Short Title | Etiologic Diagnosis of LRTI via LAMP |
| Protocol Number | 00001384 |
| Study design | Consecutive LRTI patients are planed to be enrolled in multi-centers, sputum samples from eligible subjects are planed to be applied to parallel tests of qLAMP and culture/serological test, the results and accuracy of both tests are to be compared and determined statistically. |
| Study Duration | Dec 2007-Dec 2008 |
| Study Center(s) | 21 centers—21 tertiary hospitals over 14 provinces of P. R. China agreed to participate. |
| Objectives | Evaluate the accuracy of qLAMP in etiology diagnosis of LRTI patients. |
| Number of Subjects | About 3000 LRTI patients |
| Diagnosis and Main Inclusion Criteria | Patients were suspected to have bacterial LRTI with typical diagnoses of pneumonia or bronchitis based on chest X-ray, as well as clinical symptoms and simple laboratory signs. |
| The evaluated test | Quantitative LAMP of 11 common bacteria in LRTI subjects. |
| Reference test | Routine bacterial culture/serological test |
| Statistical Methodology | Using SPSS version 17.0, the data were summarized in a receiver operating characteristic (ROC) curve to determine the diagnostic efficacy, the area under the curve (AUC) and its 95% confidence interval (95% CI) were calculated as a measure for the discriminative efficacy of the index test.  R software will be used to construct statistical model to explore to relationship between the result of both tests, and calculate cut-offs if necessary. |

# Introduction

This document is a protocol for a human research study. This study is to be conducted according to international standards of Good Clinical Practice, applicable government regulations and Institutional research policies and procedures.

## Background

Lower respiratory tract infections (LRTI) are a public health problem of major concern, which account for 12% & 16% of the inpatients in city and county hospitals of China. Beyond this, LRTI also importantly contribute to antibiotic overuse and allocation of health resources worldwide. . Many LRTI are caused by organisms that do not require antibiotics or could be safely treatment with narrow-spectrum antibiotics. Identifying the etiology of LRTI by traditional culture test assay needs at least 48-72hr. And there are a large proportion of cases with no pathogen identifying, either because the appropriate tests were not performed or the organism was missed. Thus, treatment of LRTI is generally empirical, with physicians reliant on previous etiology and microbiology studies to guide their choice of intervention, although each of these can be misleading. It is necessary to explore new, rapid and accurate diagnostic techniques.

A PCR-Chip assay for sepsis named prove-itTM was approved in Finland, which detects 31 pathogen bacteria in 3hours. But this non-quantitative method only work with blood or body fluid normally asepsis, while not sputum, the most convenient and routine specimen of LRTI, as many other similar methods . Real-time PCR, NASBA, LAMP, and many quantitative nuclear acid amplification methods had been successively applied in clinical virus detection, such as CMV, HCV, HBV, HIV. And some of these methods had been approved to be able to detect several pathogens simultaneously with perfect sensitivity and specificity in standard bacteria stains. While in the term of bacteria or fungi, these methods showed much more positive results in clinical sputum sample tests according to traditional culture assay. The discrepancies must be rationally explained before these methods to be applied in clinical.

## Index test

Loop mediated isothermal amplification (LAMP) is a single tube technique for the amplification of [DNA](http://en.wikipedia.org/wiki/DNA)[[1]](http://en.wikipedia.org/wiki/Loop-mediated_isothermal_amplification#cite_note-0%23cite_note-0). It may be combined with a [reverse transcription](http://en.wikipedia.org/wiki/Reverse_transcription) step to allow the detection of RNA. LAMP is a novel approach to nucleic acid amplification which uses a single temperature incubation thereby obviating the need for expensive [thermal cyclers](http://en.wikipedia.org/wiki/Thermal_cyclers). Detection of amplification product can be by photometry for turbidity caused by increasing quantity of Magnesium pyrophosphate in solution [[2]](http://en.wikipedia.org/wiki/Loop-mediated_isothermal_amplification#cite_note-1%23cite_note-1) or with addition of [SYBR green](http://en.wikipedia.org/wiki/SYBR_green), a color change can be seen without equipment.

The reaction can be followed in real-time either by measuring the turbidity [3] or the signals from DNA produced via fluorescent dyes that intercalate or directly label the DNA, and in turn can be correlated to the number of copies initially present. Hence, LAMP can also be quantitative. While LAMP is widely being studied for detecting infectious diseases such as tuberculosis, malaria [4], and sleeping sickness in developing regions, it has yet to be extensively validated for other common pathogens.

## Preclinical Data

Genomic sequences of 11 bacteria species, i.e. *Acinetobacter baumannii, Escherichia coli, Haemophilus influenzae, Klebsiella pneumoniae, Pseudomonas aeruginosa, Staphylococcus aureus, Stenotrophomonas maltophilia, Streptococcus pneumoniae*, *Legionella pneumophila, Mycoplasma pneumoniae* and *Chlamydophila pneumoniae* were retrieved from Genebank. Each primer set was designed based on the strategy described previously, and sensitivity, specificity and reproducibility of them were evaluated by quantified DNA from 27 species of bacteria. Quantitative LAMP assay was performed by real-time fluorescence detection modified from a previously published protocol.

Quantified DNA of the 11 tested bacteria, which was extracted and purified by QIAamp according to the manufacturer’s instructions, was serially diluted as positive control. Concentration of copy-number of genomic equivalents was calculated based on its genome size to plot standard curve. Then the equivalent copy numbers of the template DNA were determined based on the standard curves generated from quantified control DNA.

The sensitivity of each set of primers was determined by serially diluted each targeted DNA template as the consistently detectable minimal content (copies/μl DNA template), then traced to the titer of bacteria in sputum by the following equation:

Titer in sputum [copies/ml] = copies/μl (content in DNA template) × 100μl (template volume) / 0.5 ml (sputum volume)

The specificity of each set of primers were evaluated by quantified bacteria DNA of 27 species (8 targeted and 19 reference species, listed in Supplement Table 4) obtained as mentioned above, to exclude primer set show any cross reaction with other species DNA.

Serially diluted each targeted DNA templates were tested at least 8 times at different time and on different machines. The ranges of error were calculated to evaluate the reproducibility of our LAMP system.

Our primer sets for the 11 bacteria showed sensitivities to a titer of 10^3^copies/ml in sputa and specificities where no detectable cross-reaction with DNA from 26 other bacterial species. The maximum range of titer from single standard DNA temple was less than 6 fold. These showed acceptable sensitivity, specificity and reproducibility of our LAMP system.

# Study Objectives

Primary Objective

To compare and determine the etiology diagnostic test accuracy of qLAMP test and routine bacteria culture/serological test for the etiology diagnosis in LRTI patients with sputum samples.

Secondary Objective

1. To compare results of qLAMP with the results of routine culture/serological test, or

Assess the relationship between the results of qLAMP and routine bacterial culture/serological test.

2. To evaluate the necessity of quantization and cut-off setting.

3. To explore heterogeneity in term of bacterial pathogens involved in LRTI.

# Subject Selection

## Inclusion Criteria

Patients were suspected to have bacterial LRTI with typical diagnoses of pneumonia or bronchitis based on chest X-ray, as well as one of the following criteria:

(1) fever>38.5^0^C,

(2) peripheral white blood cell count (WBC)>10.0×10^9^/L and neutrophil % >60%,

(3) exacerbated dyspnea

(4) exacerbated sputum production or purulence.

## Exclusion Criteria

After informative diagnosis, patients rectified as one of the following:

(1) non-infectious diseases

(2) mainly infected by virus,

(3) mainly infected by fungi,

(4) mainly infected by pneumocystis

(5) mainly infected by TB

## Subject Recruitment and Screening

Potential bacterial LRTI patients were consecutively enrolled from 21 tertiary hospitals over 14 provinces in People’s Republic of China as the inclusion criteria, then samples will be collected to quality evaluation. Subjects with qualified samples will be applied to qLAMP and routine culture/serological test assay. Subjects adapt to exclusion criteria after informative diagnosis will be excluded.

# Study Design and Procedures

The study will be conducted according to the following design and procedures in the flowchat:

Exclusion by no qualified sample

Exclusion by rectified diagnosis as non-infectious diseases or non-bacterial infections

Specimen collection and evaluation

men

ollection and evaluation

Consecutive suspected LRTI patients according to inclusion criteria

Informed Consent Form Signed

Clinical information input

Statistical analysis

1^st^ day anticoagulated blood

1^st^ day

sputum

1^st^ day

sputum

3^r^ day

sputum

2^nd^ day

sputum

anticoagulated blood (at least 10days late)

)

LAMP & Culture in core lab

Culture in local hospital

Serological test in core lab

# Statistical Plan

## Sample Size

Sample size of this study will not be accurately estimated because lack of accurate positive rate for each evaluated pathogen caused infection in LRTI patients. We estimate the positive rates range 5-15%, and the sensitivity of the index qLAMP test is 90- 95%, then 400-3000 eligible patients are needed for statistical analysis. We finally plan to enroll 1500 eligible patients..

## Statistical Methods

Using SPSS version 17.0, the data were summarized in a receiver operating characteristic (ROC) curve to determine the diagnostic efficacy, the area under the curve (AUC) and its 95% confidence interval (95% CI) were calculated as a measure for the discriminative efficacy of the index test.

R software will be used to construct statistical model to explore to relationship between the result of both tests, and calculate cut-offs if necessary.

## Subject Population(s) for Analysis

the subject populations whose data will be subjected to the study analysis is the population, in which any subject who was randomized and had qualified samples and clinical informations.

# Safety and Adverse Events

This protocol will only collect vein blood (once or twice) and spontaneous sputum samples in most cases. For the subjects who can not collect spontaneous sputum, only those applied to fiberoptic bronchoscopy or mechanical ventilation according to clinical requirement, through which sputa or BALF samples can be obtained, would be enrolled.

So theoretically there would not be any adverse events related to the application of the protocol.

# Data Handling and Record Keeping

## Confidentiality

Information about study subjects will be kept confidential and managed according to the requirements of the Institute, which require a signed subject authorization informing the subject, who will have access to that information and why, and who will use or disclose that information.

## Source Documents

Source data is all information, original records of clinical findings, observations, or other activities in a clinical trial necessary for the reconstruction and evaluation of the trial. Source data are contained in source documents. Examples of these original documents, and data records include: hospital records, clinical and office charts, laboratory notes, memoranda, subjects’ diaries or evaluation checklists, pharmacy dispensing records, recorded data from automated instruments, copies or transcriptions certified after verification as being accurate and complete, microfiches, photographic negatives, microfilm or magnetic media, x-rays, subject files, and records kept at the pharmacy, at the laboratories, and at medico-technical departments involved in the study.

## Records Retention

It is the investigator’s responsibility to retain study essential documents for at least 5 years after the end of the study. These documents should be retained for a longer period if required by an agreement with the sponsor. In such an instance, it is the responsibility of the sponsor to inform the investigator/institution as to when these documents no longer need to be retained.

# Study Monitoring, Auditing, and Inspecting

## Study Monitoring Plan

This study will be monitored according to the monitoring plan in Attachment 1. The investigator will allocate adequate time for such monitoring activities. The Investigator will also ensure that the monitor or other compliance or quality assurance reviewer is given access to all the above noted study-related documents and study related facilities (e.g. diagnostic laboratory), and has adequate space to conduct the monitoring visit.

## Auditing and Inspecting

The investigator will permit study-related monitoring, audits, and inspections by the EC/IRB, the sponsor, government regulatory bodies, and University compliance and quality assurance groups of all study related documents (e.g. source documents, regulatory documents, data collection instruments, study data etc.). The investigator will ensure the capability for inspections of applicable study-related facilities (e.g. diagnostic laboratory).

Participation as an investigator in this study implies acceptance of potential inspection by government regulatory authorities and applicable University compliance and quality assurance offices.

# Ethical Considerations

This study is to be conducted according to P.R.China and international standards of Good Clinical Practice, applicable government regulations and Institutional research policies and procedures.

This protocol and any amendments will be submitted to the Institutional Review Board (IRB), in agreement with local legal prescriptions, for formal approval of the study conduct. The decision of the IRB concerning the conduct of the study will be made in writing to the investigator and a copy of this decision will be provided to the sponsor before commencement of this study.

All subjects for this study will be provided a consent form describing this study and providing sufficient information for subjects to make an informed decision about their participation in this study. See Attachment 3 for a copy of the Subject Informed Consent Form. This consent form will be submitted with the protocol for review and approval by the IRB for the study. The formal consent of a subject, using the IRB-approved consent form, must be obtained before that subject undergoes any study procedure. The consent form must be signed by the subject or legally acceptable surrogate, and the investigator-designated research professional obtaining the consent.

# Study Finances

## Funding Source

This study is financed through a grant from the Ministry of Science and Technology of P.R.China. (National High-Tech R&D program: 2006AA02Z4A9)

## Conflict of Interest

Any investigator who has a conflict of interest with this study (patent ownership, royalties, or financial gain greater than the minimum allowable by their institution, etc.) must have the conflict reviewed by a properly constituted Conflict of Interest Committee with a Committee-sanctioned conflict management plan that has been reviewed and approved by the study sponsor prior to participation in this study.

# Publication Plan

Neither the complete nor any part of the results of the study carried out under this protocol, nor any of the information provided by the sponsor for the purposes of performing the study, will be published or passed on to any third party without the consent of the study sponsor. Any investigator involved with this study is obligated to provide the sponsor with complete test results and all data derived from the study.

# References

1. Notomi T, etal 2000. Loop-mediated isothermal amplification of DNA. Nucleic Acids Research 28:E63
2. Mori, Y., Nagamine, K., Tomita, N., Notomi, T. (2001). Detection of loop-mediated isothermal amplification reaction by turbidity derived from magnesium pyrophosphate formation. Biochemical and Biophysical Research Communications 289: 150-154.
3. Mori, Y., Kitao, M., Tomita, N., Notomi, T. (2004). Real-time turbidimetry of LAMP reaction for quantifying template DNA. Journal of Biochemical and Biophysical Methods 59: 145-157.
4. Poon, L.L.M., Wong, B.W.Y., Ma, E.H.T., Chan, K.H., Chow, L.M.C., Abeyewickreme, W., Tangpukdee, N., Yuen, K.Y., Guan, Y., Looareesuwan, S., Peiris, J.S.M. (2006). Sensitive and inexpensive molecular test for falciparum malaria: detecting Plasmodium falciparum DNA directly from heat-treated blood by loop-mediated isothermal amplification. Clinical Chemistry 52: 303-306.

# Attachments (have not translated into English)

1. Study Monitoring Plan
2. Investigator Agreement
3. Sample Consent Form
4. Specimen Collection, Preparation & Handling (SOP)
5. Instruction For On-Line Database Input
